# Effect of E-Vaping on Kidney Health in Mice Consuming a High-Fat Diet

**DOI:** 10.3390/nu15143140

**Published:** 2023-07-14

**Authors:** Min Feng, Xu Bai, Andrew E. Thorpe, Long The Nguyen, Meng Wang, Brian G. Oliver, Angela S. Y. Chou, Carol A. Pollock, Sonia Saad, Hui Chen

**Affiliations:** 1 School of Life Sciences, Faculty of Science, University of Technology Sydney, Sydney, NSW 2007, Australia; min.feng@student.uts.edu.au (M.F.); xu.bai@student.uts.edu.au (X.B.); andrew.e.thorpe@student.uts.edu.au (A.E.T.); meng.wang-12@student.uts.edu.au (M.W.); brian.oliver@uts.edu.au (B.G.O.); 2 Respiratory Cellular and Molecular Biology, Woolcock Institute of Medical Research, Macquarie University, Glebe, NSW 2037, Australia; 3 Renal Group, Kolling Institute of Medical Research, The University of Sydney, St Leonards, NSW 2064, Australia; long.t.nguyen@sydney.edu.au (L.T.N.); carol.pollock@sydney.edu.au (C.A.P.); 4 NSW Health Pathology, Royal North Shore Hospital, The University of Sydney, St Leonards, NSW 2064, Australia; angelashihyuan.chou@health.nsw.gov.au

**Keywords:** inflammation, oxidative stress, nicotine, nicotine-free, e-cigarette, fibrosis

## Abstract

High-fat diet (HFD) consumption and tobacco smoking are risk factors for chronic kidney disease. E-cigarettes have gained significant popularity among younger populations worldwide, especially among overweight individuals. It is unclear whether vaping interacts with HFD consumption to impact renal health. In this study, Balb/c mice (male, 7 weeks old) were fed a pellet HFD (43% fat, 20 kJ/g) for 16 weeks when exposed to nicotine or nicotine-free e-vapour from weeks 11 to 16. While HFD alone increased collagen Ia and IV depositions, it did not cause significant oxidative stress and inflammatory responses in the kidney itself. On the other hand, e-vapour exposure alone increased oxidative stress and damaged DNA and mitochondrial oxidative phosphorylation complexes without significant impact on fibrotic markers. However, the combination of nicotine e-vapour and HFD increased inflammatory responses, oxidative stress-induced DNA injury, and pro-fibrotic markers, suggesting accelerated development of renal pathology. Nicotine-free e-vapour exposure and HFD consumption suppressed the production of mitochondrial OXPHOS complexes and extracellular matrix protein deposition, which may cause structural instability that can interrupt normal kidney function in the future. In conclusion, our study demonstrated that a HFD combined with e-cigarette vapour exposure, especially when containing nicotine, can increase susceptibility to kidney disease.

## 1. Introduction

Chronic kidney disease is a pressing global health concern, affecting 8–18% of the population worldwide. The prevalence of this disease is closely associated with the global rise in obesity and diabetes rates. Overconsumption of diets high in fat and sugar has led to an ongoing pandemic of obesity, which has been well studied for its direct and adverse effects on kidney health using rodent models by ourselves and others [1]. Obesity carries inherent risks for type 2 diabetes, which in turn can contribute to the onset of nephropathy—a condition that damages the integrity of the glomeruli, results in inflammatory responses and tubulointerstitial fibrosis and impairs kidney function. Importantly, the pre-diabetic status that arises from long-term consumption of a high fat diet (HFD) can pose similar risks to kidney health. In previous studies, inflammation and oxidative stress have been identified as major factors in developing chronic kidney disease in rodent models, leading to fibrosis, loss of basement membrane integrity, and tubular and glomerular injury [1,2,3]. These structural changes inevitably result in renal dysfunction and progressive chronic kidney disease.

The kidney is also highly susceptible to environmental toxins [4,5,6,7]. The role of tobacco smoking as a prominent risk factor in the development of chronic kidney disease is widely recognised, encompassing both direct and indirect impacts on renal health [8,9,10]. Over the past few years, there has been a notable surge in the popularity of e-cigarettes among younger individuals, partly due to their appeal as a trendy device and as a “safe cigarette”, which has fewer toxicants and thus presumed lower lung disease- and cancer-producing effects compared with traditional tobacco cigarettes. While the e-cigarette industry promotes these devices as aids for smoking cessation and the health authorities also strictly recommend them for such purposes, their predominant usage is primarily recreational among most users, especially the young. This recreational use is driven by the availability of various flavours and the inclusion of nicotine content that results in addiction. Evidence of the link between e-vaping and metabolic disorders has emerged from human and animal studies [11]. In wild-type mice, our own study has shown increased glucose intolerance caused by nicotine-containing e-vapour exposure, while nicotine-free e-vapour exposure increased dyslipidaemia and reduced fat mass and body weight in mice fed an HFD [12]. No substantial evidence between e-vaping and increased risk of diabetes has been found in humans yet [13]. To date, there is no conclusive human evidence linking e-cigarette usage to kidney health either. This is not unexpected, given that e-cigarettes have only been on the market for a short time, and it may take several decades to establish their impact on the development of chronic diseases, including kidney disease, in humans.

E-cigarette vaping alone can cause a systemic inflammatory response and fibrotic effects in many organs, including the lungs and distal organs, such as the kidney [14,15,16,17]. Being the organ directly exposed to e-vapour, inflammatory responses owing to immune cell infiltration and the release of inflammatory cytokines lead to tissue injuries and changes in a suite of matrix metalloproteinases resulting in extracellular matrix remodelling and small airway fibrosis [18,19]. Similar pathological changes can also happen in the kidney. However, when an HFD is present, the systemic inflammatory response induced by nicotine-free e-vapour exposure is suppressed in obese mice [16]. Yet, local inflammatory and fibrotic markers are increased in the liver [12]. This suggests that such local pathological responses in specific organs, such as the liver, may be only triggered by systemic inflammation, even if the level is not as high as that under infectious conditions. It is possible that local responses may be influenced by distinct factors or mechanisms that are not solely dependent on the level of systemic inflammation, such as the infiltration of circulating immune cells. 

As a relatively new product on the market, the impacts of e-vapour inhalation on renal pathological changes are still unclear, as are the potential additive effects of HFD and e-vapour inhalation. It is noteworthy that individuals with pre-existing conditions, such as obesity and pre-diabetes, are already susceptible to underlying renal damage [20]. The combination of these factors, along with the additional effects of e-cigarette vapour exposure with or without the presence of nicotine, may exacerbate the potential impact on kidney health and increase the likelihood of developing chronic kidney disease. In addition, the presence of other toxic compounds in e-vapour generated during heating can act as an inflammatory stimulus, potentially leading to the infiltration of macrophages and the production of excess pro-inflammatory cytokines (e.g., monocyte chemoattractant protein (MCP)-1 and TNF-α) within the kidney. These cytokines are recognised for their ability to induce the production of collagens, thereby promoting the process of fibrogenesis [21,22,23,24]. Under such an inflammatory condition, mesangial cells residing in the glomerulus release inflammatory markers that initiate the infiltration of monocytes and intensify oxidative stress [25,26]. Consequently, this cascade of events triggers mitochondrial damage and dysfunction in the glomerular filtration system [26,27,28]. Therefore, it is hypothesised that in the presence of chronic HFD consumption, e-vapour exposure may exacerbate pathological damage in the kidneys. In this study, we exposed HFD-fed mice to both nicotine-containing and nicotine-free e-vapour to determine whether any effects on the renal inflammatory response, oxidative stress and fibrotic markers are dependent on the presence of nicotine.

## 2. Materials and Methods

### 2.1. Animal Experiments

All experimental procedures were conducted in accordance with the Australian National Health & Medical Research Council Code for the Care and Use of Laboratory Animals. The protocol was approved by the Animal Ethics and Care Committee at Northern Sydney Local Health District (RESP17/93).

Balb/c mouse is the most susceptible strain to cigarette smoke-induced respiratory conditions and other organ disorders, akin to those observed in human smokers, as demonstrated in our previous studies with reproducible results [15,24,29,30,31,32]. Therefore, this strain was selected for this study to model the effect of e-cigarette vapour exposure. The treatment protocol has been described in detail in a previous paper using the same animals [16]. Briefly, a commercially available pellet HFD similar to the Australian diet composition (43% fat, 20 kJ/g, SF03-20, Specialty Feeds, Glen Forrest, WA, Australia) was used to induce dietary obesity in male Balb/c mice (7 weeks of age) by feeding them for 16 weeks. A commercially available standard chow (17% fat, 13 kJ/g, Gordon’s Specialty Stockfeeds, Yanderra, NSW, Australia) was used as a control, representing a balanced diet. Mice started with similar average body weights in each group. After 10 weeks of feeding, mice in each dietary group were further divided into three sub-groups with similar average body weights. In a 19 L homemade Perspex chamber, one sub-group was exposed to nicotine-containing e-vapour (18 mg/mL; e-cig18, 50% propylene glycol/50% vegetable glycerine, tobacco flavour, Vapor Empire, VIC), one was exposed to nicotine-free e-vapour (e-cig0; 50% propylene glycol/50% vegetable glycerine, tobacco flavour, Vapor Empire, VIC), and the third sub-group was exposed to ambient air (Sham) for 30 min, twice daily in a fume hood. Nicotine and nicotine-free e-vapours were generated from 3rd generation e-cigarette devices (KangerTech NEBOX, KangerTech, Shenzhen, China) as we used in [15]. As a result, the six experimental groups were chow + sham, chow + e-cig18, chow + e-cig0, HFD + sham, HFD + e-cig18 and HFD + e-cig0. 

Twenty-four hour urine was collected using a metabolic cage at 15 weeks. At the endpoint, mice underwent deep anaesthesia using isoflurane (2%) for tissue harvesting. The left kidney was weighed and kept at −80 °C for later measurement of mRNA and proteins of interest. The right kidney was fixed in formalin for later histology analysis. Urine albumin levels were determined using the Murine Microalbuminuria ELISA kit (Exocell, Inc., Philadelphia, PA, USA), and urine creatinine levels were determined using the Microcreatinuria ELISA kit (Exocell, Inc., Philadelphia, PA, USA).

### 2.2. Real-Time PCR

mRNA expression was measured following our previously published protocols [12,28,31]. Total mRNA was extracted from kidney samples using TriZol reagent (Sigma-Aldrich, St. Louis, MO, USA) according to the manufacturer’s instructions. Tissues were homogenised using a Precellys homogeniser (Bertin Technologies, Montigny-le-bretonneux, Ile-de-France, France), followed by extraction using chloroform and isopropyl alcohol. RNA concentrations were quantified using the NanoDrop^®^ (Thermo Fisher Scientific, Waltham, MA, USA). An M-MLV Reverse Transcriptase, Rnase H, and Point Mutant Kit (Promega, Madison, WI, USA) were employed to synthesise cDNA using purified mRNA as the template. Manufactured pre-optimised Taqmen^®^ primers/probes mixtures (Thermo Fisher Scientific, Waltham, MA, USA) were used to measure the genes of interest (CFX96^TM^ Real-Time System, Bio-Rad Laboratories, Hercules, CA, USA). The probes of the housekeeping 18s RNA were labelled with VIC^®^ dye, whilst the probes of target genes were labelled with FAM^®^. The probe sequences provided by the manufacturer have been reported in our previous paper [33]. The housekeeping gene 18s rRNA was used as a reference gene, against which the target gene expression was calculated using the 2^−ΔΔCt^ method. The average level of the control group (chow + sham) was used as the calibrator, and all other samples were expressed as fold difference against it.

### 2.3. Renal Structural Changes

Formalin-fixed kidneys (*n* = 6) sectioned at 2 μm thickness underwent Hematoxilyn and Eosin (H&E) staining and were assessed using a light microscope (Leica, Wetzlar, Germany). Glomerulosclerosis index and tubulointerstitial fibrosis were assessed blindly, as we have previously described, by two independent pathologists [34].

### 2.4. Immunohistochemistry

Immunohistochemistry staining was used to measure macrophage number (F4/80), DNA oxidative stress injury (8-Hydroxyguanosine, 8OhdG) and myofibroblasts markers (α-smooth muscle actin, α-SMA). Formalin-fixed kidneys were embedded in paraffin and sectioned at 5 μm. The sections then underwent homogenisation and rehydration through a sequential treatment involving xylene and progressively decreasing concentrations of ethanol. Antigen retrieval was performed before the sections were incubated with the primary antibodies, including F4/80 (1:400, Abcam, Cambridge, UK), 8OhdG (1:200, Bioss, Rabbit, bs-1278R, Woburn, MA, USA) and α-SMA (1:200, Bio-Rad, Rat, MCA497G, Hercules, CA, USA). The positive staining was developed using the horseradish peroxidase anti-rabbit Envision system (Dako Cytochemistry, Tokyo, Japan). The sections were then counterstained with hematoxylin. Five non-overlapping areas from each animal were analysed and averaged with the Fiji software version 2.9.0 (National Institutes of Health, Bethesda, MD, USA).

### 2.5. Western Blotting

Protein levels of endogenous antioxidant manganese superoxide dismutase (MnSOD) and mitochondrial oxidative phosphorylation (OXPHOS) complexes were measured using our published methods [3,35,36]. Briefly, frozen kidney tissues (50–100 mg) were homogenised in cold lysis buffer and extracted proteins were quantified using the Bio-Rad Protein Assay Kit (Bio-Rad Laboratories, Hercules, CA, USA). The NuPage^®^ Novex^®^ 4–12% Bis-Tris gels (Thermo Fisher Scientific, Pleasanton, CA, USA) were used to separate the protein samples (40µg), and proteins in the gel were transferred to PVDF membranes (Rockford, IL, USA). The PVDF membranes were incubated with Ponceau S (15 min) to visualise the bands. The membranes were then blocked with the no-fat milk powder, followed by incubation with the primary antibodies, including MnSOD (1:2000, Millipore, Burlington, MA, USA), Mitoprofile Total^®^ OXPHOS complex Rodent Western blotting antibody (1:2500, Abcam, UK) and β-actin (1:3000, Bio-Rad Laboratories, CA, USA) overnight at 4 °C. Then, the membranes were incubated with the secondary antibodies (1:5000, goat anti-rabbit or rabbit anti-mouse IgG horseradish peroxidase conjugated secondary antibody, Santa Cruz Biotechnology, Dallas, TX, USA) for 1 h. Prior to imaging, the staining of the membrane was enhanced using the Clarity^TM^ Western Enhanced Chemiluminescence (ECL) Substrate (Bio-Rad Laboratories, Hercules, CA, USA). The detection of the bands was performed using the ChemiDoc^TM^ MP Imaging System (Bio-Rad Laboratories, Hercules, CA, USA) to capture the images. Fiji software (National Institute of Health, Bethesda, Rockville, MD, USA) was used to measure band density. β-actin was used as a housekeeping protein.

### 2.6. Statistical Methods

The results are expressed as mean ± standard error of the mean (SEM). The differences among experimental groups were analysed via two-way ANOVA followed by Fisher’s least significant difference (LSD) post hoc tests (GraphPad Prism 9, GraphPad, San Diego, CA, USA). The difference was considered statistically significant if *p* < 0.05.

## 3. Results

### 3.1. Effects on Body Weight and Kidney Mass

HFD consumption significantly increased body weight and nearly doubled abdominal fat mass in sham exposed mice (*p* < 0.05 and *p* < 0.001, respectively, HFD + sham vs. chow + sham groups). However, kidney masses as net weight and percentage of body weight were not significantly affected by HFD feeding (Table 1).

E-vapour exposure led to a smaller body weight independent of nicotine content in chow-fed mice (*p* < 0.05, both chow + e-cig18 and chow + e-cig0 vs. chow + sham groups). In contrast, in HFD-fed mice, only nicotine-free e-vapour exposure significantly reduced body weight (*p* < 0.05, HFD + e-cig0 vs. HFD + sham groups, Table 1). Kidney-to-body weight (%) in HFD + e-cig0 mice was much bigger than that in the HFD + sham mice due to smaller body weight (*p* < 0.05, Table 1). There was a trend of e-vapour to reduce abdominal fat mass in chow-fed mice, more marked with nicotine-free e-vapour. Although the fat mass was smaller in HFD + e-cig0 group (*p* < 0.05 vs. HFD + sham group), the significance disappeared when it was standardised by body weight (Table 1).

### 3.2. Effect on Urinary Markers of Kidney Damage

There were no significant changes in urinary albumin/creatinine ratio in mice exposed to HFD alone (HFD + sham vs. chow + sham groups). However, there was a significant difference in urinary albumin/creatinine ratio between the chow + e-cig18 and chow + sham groups (*p* < 0.001, Table 1), but not between chow + e-cig0 and chow + sham groups. In the mice consuming an HFD, neither nicotine-containing e-vapour nor nicotine-free e-vapours affected the urinary albumin/creatinine ratio compared with the HFD + sham mice (*p* < 0.01, HFD + e-cig18 vs. chow + e-cig18 mice, Table 1).

### 3.3. Effects on Inflammatory Responses

**Figure 1:**
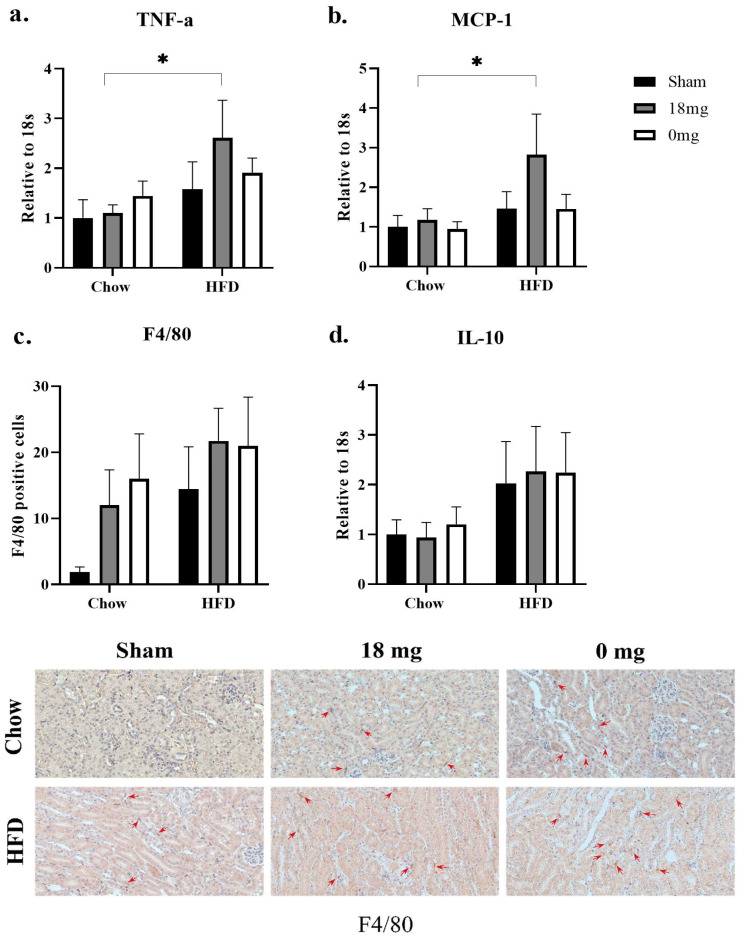
Inflammatory markers TNFa mRNA expression (**a**), MCP-1 mRNA expression (**b**), F4/80 positive cells (**c**) and representative images (red arrows indicating positive staining, 40×) of IL-10 mRNA expression (**d**). The results are expressed as mean ± SEM, *n* = 5–6. * *p* < 0.05.

HFD consumption did not significantly change the pro-inflammatory markers measured in this study, including TNFα and MCP-1 expression in the HFD + sham mice (Figure 1a,b). F4/80 counts were much higher in the HFD + sham group compared with the chow + sham group, albeit with no statistical significance (Figure 1c). The levels of the anti-inflammatory cytokine IL-10 were also doubled in the HFD + sham mice compared with the chow + sham mice, although without statistical significance (Figure 1d). 

Exposure to nicotine-containing e-vapour more than doubled renal TNFα and MCP-1 expression in HFD-fed mice, compared with their chow-fed counterparts (*p* < 0.05, HFD + e-cig18 vs. chow + e-cig18 groups, Figure 1a,b). Both nicotine-containing (*p* = 0.07, chow + e-cig18 vs. chow + sham groups) and nicotine-free (*p* = 0.06, chow + e-cig0 vs. chow + sham groups) e-vapour exposure showed a trend of increasing F4/80 positive cell counts in chow-fed mice (Figure 1c). In the HFD-fed mice, e-vapour exposure did not induce a marked change in the number of F4/80 positive cells compared with HFD + sham group (Figure 1c). IL-10 expression was not affected by e-vapour exposure in mice consuming a HFD diet, regardless of nicotine content (Figure 1d).

### 3.4. Effects on Oxidative Stress Markers and Mitochondrial Functional Units

**Figure 2:**
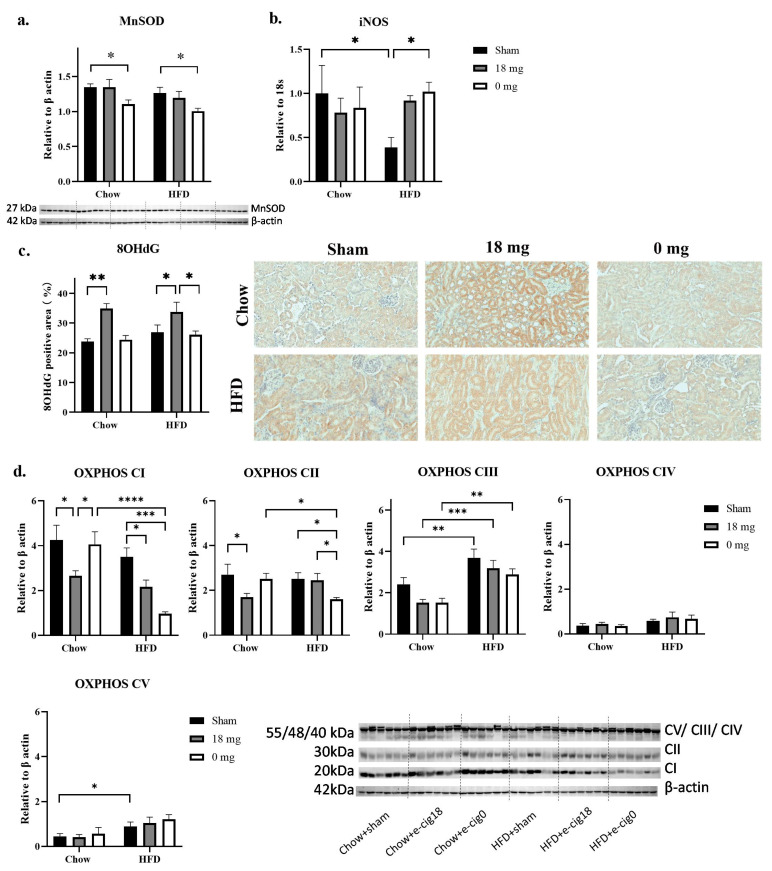
Oxidative stress markers MnSOD protein (**a**), iNOS mRNA (**b**), percentage of 8OHdG positive area (**c**) and mitochondrial functional marker OXPHOS CI-V (**d**). The results are expressed as mean ± SEM, *n* = 5–6. * *p* < 0.05, ** *p* < 0.01, *** *p* < 0.001, **** *p* < 0.0001.

HFD consumption alone did not significantly change endogenous antioxidant MnSOD levels in HFD + sham mice (Figure 2a) but reduced oxidative stress marker inducible nitric oxide synthase (iNOS) mRNA expression (*p* < 0.05 HFD + sham vs. chow + sham groups, Figure 2a). It did not affect DNA injury marker 8OhdG staining (Figure 2c), while OXPHOS CIII and V levels were increased in the HFD + sham group (*p* < 0.01 and *p* < 0.05, respectively, HFD + sham vs. chow + sham groups, Figure 2d).

Exposure to nicotine-containing e-vapour increased 8OHdG staining in both chow and HFD-fed mice (*p* < 0.01 chow + e-cig18 vs. chow + sham groups; *p* < 0.05 HFD + e-cig18 vs. HFD + sham groups, Figure 2c). It also significantly reduced OXPHOS CI levels in both chow and HFD-fed mice, but only reduced OXPHOS CII levels in chow-fed mice (*p* < 0.05 chow + e-cig18 vs. chow + sham groups, Figure 2d). OXPHOS CIII levels showed a trend of decrease in the chow + e-cig18 mice compared with the chow + sham group (Figure 2d).

Exposure to nicotine-free e-vapour reduced MnSOD levels in both chow and HFD-fed mice (*p* < 0.05 chow + e-cig0 vs. chow + sham groups, HFD + e-cig0 vs. HFD + sham groups, Figure 2a). It increased iNOS mRNA expression in HFD + e-cig0 mice (*p* < 0.05 HFD + e-cig0 vs. HFD + sham groups, Figure 2b), but suppressed OXPHOS CI and CII levels in HFD-fed mice only (*p* < 0.001 and *p* < 0.05, respectively, HFD + e-cig0 vs. HFD + sham groups, Figure 2d). OXPHOS CIII levels were reduced in the chow + e-cig0 mice compared with the chow + sham group, although this did not reach significance (Figure 2d).

### 3.5. Effect on Fibrotic Markers

**Figure 3:**
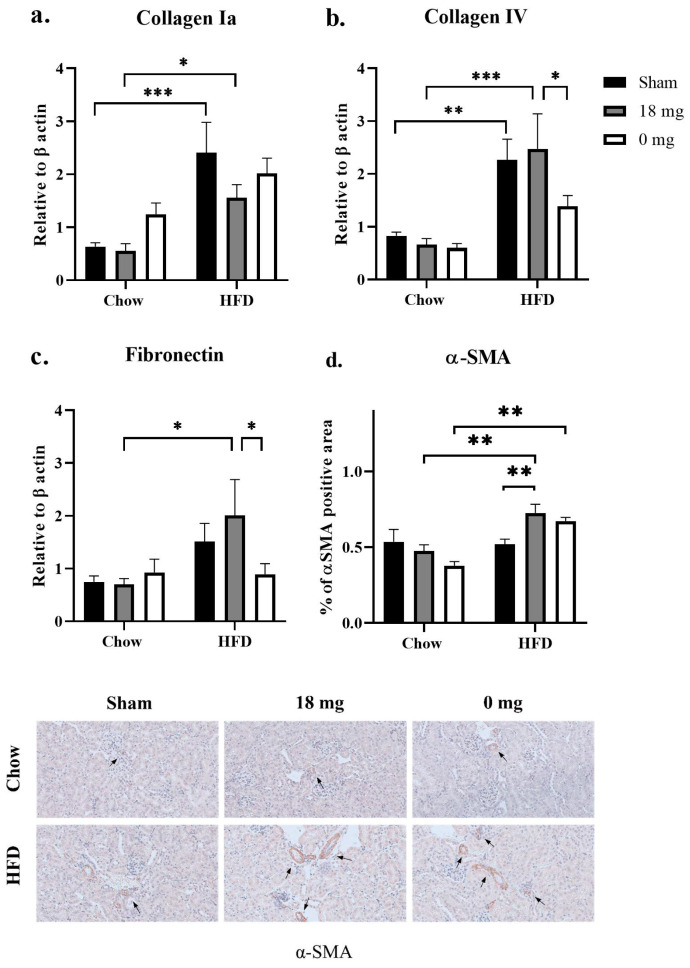
mRNA expression of fibrotic markers collagen Ia (**a**), collagen IV (**b**), fibronectin (**c**), and percentage of α-SMA positive area (**d**) marked with black arrows in IHC staining (e, 40×). The results are expressed as mean ± SEM, *n* = 5–6. * *p* < 0.05, ** *p* < 0.01, *** *p* < 0.001.

HFD consumption significantly increased collagen Ia and IV (*p* < 0.001 and *p* < 0.01, respectively, HFD + sham vs. chow + sham groups; *p* < 0.05 and *p* < 0.001, respectively, HFD + e-cig18 vs. chow + e-cig18 groups; Figure 3a,b). Fibronectin expression was increased by 50% in HFD-fed mice (HFD + sham vs. chow + sham groups), albeit without statistical significance (Figure 3c). αMSA protein expression did not change as a result of HFD consumption (Figure 3d).

HFD consumption increased levels of fibrotic markers in the absence or/and presence of e-cigarette vapour exposure. Exposure to nicotine-containing e-vapour makes the HFD-diet effect significant between HFD + e-cig18 and chow + e-cig18 (*p* < 0.05, Figure 3c). Compared with HFD + e-cig18 mice, levels of collagen IV and fibronectin were lower in HFD + e-cig0 mice (*p* < 0.05, Figure 3b,c). Nicotine-containing e-vapour exposure also significantly increased αSMA staining in HFD-fed mice (*p* < 0.05, HFD + e-cig18 vs. HFD + sham; *p* = 0.057, HFD + e-cig0 vs. HFD + sham groups; *p* < 0.01, HFD + e-cig18 vs. chow + e-cig18 groups, HFD + e-cig0 vs. chow + e-cig0 groups; Figure 3d). There were no significant changes in tubulointerstitial fibrosis scores between the groups (data not shown).

## 4. Discussion

The major finding of this study is that interaction between HFD consumption and nicotine-containing e-vapour exposure increased inflammatory responses, oxidative stress-induced DNA injury and pro-fibrotic markers, suggesting accelerated development of renal fibrosis. There was also an interaction between HFD consumption and nicotine-free e-vapour exposure in suppressing the production of mitochondrial OXPHOS complexes I and II, as well as extracellular matrix proteins, which may cause structural instability that could interrupt normal kidney function in the future.

In this study, nicotine-containing e-vapour seems more detrimental to kidney health than nicotine-free e-vapour, reflected by increased renal urinary albumin/creatinine ratio. Despite no significant changes in the inflammatory cytokines measured in chow + e-cig18 mice, with increased macrophage number (reflected by F4/80 staining), nicotine-containing e-vapour alone still caused more oxidative stress-induced DNA damage and impaired mitochondrial functional units where the energy substrate enters the OXPHOS chain for ATP synthesis. This may suggest that other, unknown mediators produced by macrophages may contribute, which could be followed up in future studies. Nicotine-containing e-vapour in the presence of HFD increased inflammatory responses, oxidative stress-induced DNA injury, abnormal mitochondrial functional units and the activation of fibrotic signalling, which are all known to induce kidney damage.

Although HFD consumption alone caused excess collagen Ia and IV deposition and some increase in fibronectin as expected, all the above mentioned injury-triggering markers in the kidney were not significantly activated in the HFD + sham group. The excess deposition of extracellular matrix proteins in HFD + sham mice may be due to increased circulating pro-inflammatory cytokines shown in the same mice in a previous paper [16], not the local environment in the kidney itself. Nevertheless, when HFD and nicotine-containing e-vapour are combined, increased MCP-1 can serve as the initial step or trigger, which can respond to both HFD consumption and e-vaping [37,38]. MCP-1 induces the inflammatory phenotype of macrophages which release more pro-inflammatory cytokines, such as TNFα, which in turn induces renal fibrotic reactions [39,40]. Inflammation can also cause oxidative stress and related tissue injuries, reflected in the marker of DNA damage, 8OHdG, which can also directly damage mitochondria, the powerhouse of all cells. However, the impacts on DNA and mitochondria seem to be diet-independent, suggesting vaping nicotine-containing e-cigarettes may have a detrimental impact on kidney health, regardless of the user’s diet.

Nevertheless, fibrosis is the final stage of the pathological process in chronic kidney disease, with an excessive accumulation of matrix components (e.g., collagen IV). Here, a significant increase in collagen deposition was only apparent in HFD-fed mice, whereas the increase in α-SMA was only observed when HFD and e-vapour exposure were combined, suggesting additive effects on fibroblast activation. Although there were no significant changes in tubulointerstitial score in kidney tissues at this stage, increased α-SMA can continue to activate fibrogenic cells and myofibroblasts in forming fibrosis given sufficient time, i.e., further enhancing the levels of collagen Ia and fibronectin. Therefore, these findings suggest that although long term exposure to an unhealthy diet (i.e., HFD) is more potent in inducing extracellular matrix protein deposition, the additional vaping of nicotine e-cigarettes, even for a short period of time, may accelerate the structural abnormality and make the kidney more vulnerable. Indeed, a previous study exposing mice to nicotine e-vapour for 3 months showed reduced renal filtration and increased renal fibrosis due to systemic inflammation [17]. Therefore, future studies can extend the e-vapour exposure time to determine whether the exposure duration is a factor in the interaction between HFD and e-vapour exposure.

Conversely, nicotine-free e-vapour seems to be more potent in reducing endogenous antioxidant MnSOD levels than nicotine-containing e-vapour, which may be due to increased oxidative stress triggered by high levels of circulating pro-inflammatory cytokines observed in the same mice published in our previous paper [16]. The overconsumption of MnSOD by free radicals may explain fewer injury markers due to oxidative stress in mice exposed to nicotine-free e-vapour. However, oxidative stress due to weakened mitochondrial antioxidant capacity is a critical contributor to renal diseases [26]. Reduced OXPHOS complexes I and II may also reflect a direct injury due to oxidative stress, which the mitochondria are very vulnerable to. These two OXPHOS complexes play key roles in facilitating ATP production in the mitochondria, acting as the primary and secondary entry points of electrons in the respiratory chain. As the kidney is a highly metabolic organ and thus in significant energy demand, reduced OXPHOS complexes I and II can directly affect ATP production, which is fundamental to normal cellular function. In addition, e-vaping has been found to suppress tricarboxylic acid cycle activity, resulting in reduced metabolites in the blood [41]. Although it is unclear whether circulating changes can reflect similar cellular changes in the kidney, reduced metabolic substrates may also affect OXPHOS complexes delivering ATP to maintain normal cellular functions. Here, nicotine-free e-vapour showed fewer adverse effects than nicotine-containing e-vapour exposure, especially for some renal pathology markers in the setting of long-term consumption of HFD. This somehow confirmed the detrimental impact of nicotine itself on renal health, which is exaggerated when combined with an unhealthy diet [42,43]. However, nicotine-free e-vapour still showed a detrimental effect on the mitochondrial functional unit and overall renal extracellular matrix integrity, which is fundamental for the scaffolding of renal structure, given collagen IV is one of the main macromolecules in the glomerular basement membrane. Therefore, vaping both nicotine-containing and nicotine-free e-cigarettes can potentially carry an increased risk of developing kidney diseases.

There are several limitations to this study. Firstly, although our study utilised a commercially available e-fluid of a popular flavour (i.e., tobacco flavour), it is important to acknowledge that we only examined one specific brand. Additionally, the daily exposure dose of e-fluid employed in our study was relatively low, and the duration of the study was also relatively short. Future investigations could explore the effects of higher doses of e-fluid with prolonged exposure periods, potentially leading to the manifestation of more severe renal pathology. Secondly, it is crucial to recognise that while Balb/c mice are susceptible to the adverse effects of tobacco smoking, they do not possess an inherent predisposition to obesity-related conditions. Therefore, in future studies, alternative mouse strains, such as C57BL/6, which are more prone to developing metabolic disorders upon consumption of a diet high in fat and sugar, could be used. This would enable a more comprehensive examination of the effects of e-cigarette exposure in the context of obesity-related renal complications. Nevertheless, despite these limitations, our findings support the need for further research concerning the impact of e-cigarette use on human individuals. This study serves as a foundation for subsequent investigations aimed at unravelling the intricate effects of e-cigarettes on renal health, ultimately contributing to a more comprehensive understanding of the potential risks associated with e-cigarette usage in human populations. In addition, interventions to suppress inflammatory response and oxidative stress can also be considered in future studies, which may provide some protection to the kidneys and other organs in e-cigarette users [44].

## 5. Conclusions

In conclusion, our study demonstrated that the combination of HFD and e-cigarette vapour exposure, especially if nicotine-containing, can increase susceptibility to adverse kidney outcomes. This effect is mediated through elevated inflammatory responses, oxidative stress-induced injury and accelerated fibrotic changes in the kidney. Nicotine-containing e-vapour is more harmful than nicotine-free e-vapour regarding renal health. Our findings highlight the need to ban e-cigarettes to protect kidney health.

## Figures and Tables

**Table 1:** Body weight and kidney weight of the mice at the endpoint.

	Chow + Sham	Chow + e-cig18	Chow + e-cig0	HFD + Sham	HFD + e-cig18	HFD + e-cig0
**BW (g)**	27.7 ± 0.27	26.2 ± 0.51 #	25.62 ± 0.28 #	29.1 ± 0.53 *	28.9 ± 0.74 **	27.6 ± 0.45 **#
**Kidney (g)**	0.26 ± 0.0073	0.24 ± 0.008	0.24 ± 0.0066	0.27 ± 0.0055	0.27 ± 0.0080 **	0.27 ± 0.0095 **
**Kidney%**	0.93 ± 0.022	0.90 ± 0.019	0.94 ± 0.024	0.91 ± 0.023	0.94 ± 0.033	0.99 ± 0.030 #
**Abdominal Fat (g)**	0.69 ± 0.04	0.56 ± 0.07	0.52 ± 0.03	1.27 ± 0.12 ***	1.16 ± 0.13 ***	1.04 ± 0.58 ***#
**Abdominal fat%**	2.50 ± 0.17	2.14 ± 0.23	2.05 ± 0.11	4.28 ± 0.35 ***	4.04 ± 0.40 ***	3.78 ± 0.20 ***
**Albumin/** **Creatinine ratio**	27.5 ± 1.79	36.9 ± 3.67 ##	30.1 ± 2.80	26.6 ± 1.92	26.4 ± 2.05 **	24.3 ± 1.97

The results are expressed as mean ± SEM, *n* = 14–15. * *p* < 0.05, ** *p* < 0.01, *** *p* < 0.001 vs. chow-fed mice with the same treatment; # *p* < 0.05, ## *p* < 0.01 vs. sham exposed mice fed the same diet.

## Data Availability

All datasets are available upon request. All information used in this study is publicly available and referenced in the Section 2.
